# Structural Integrity of an Electron Beam Melted Titanium Alloy

**DOI:** 10.3390/ma9060470

**Published:** 2016-06-14

**Authors:** Robert Lancaster, Gareth Davies, Henry Illsley, Spencer Jeffs, Gavin Baxter

**Affiliations:** 1 Institute of Structural Materials, Swansea University, Bay Campus, Fabian Way, Swansea SA1 8EN, UK; 715624@swansea.ac.uk (G.D.); 634579@swansea.ac.uk (H.I.); s.p.jeffs@swansea.ac.uk (S.J.); 2 Rolls-Royce plc, P.O. Box 31, Derby DE24 8BJ, UK; gavin.baxter@rolls-royce.com

**Keywords:** electron beam melting, titanium alloys, small punch test, X-ray computed tomography, failure analysis

## Abstract

Advanced manufacturing encompasses the wide range of processes that consist of “3D printing” of metallic materials. One such method is Electron Beam Melting (EBM), a modern build technology that offers significant potential for lean manufacture and a capability to produce fully dense near-net shaped components. However, the manufacture of intricate geometries will result in variable thermal cycles and thus a transient microstructure throughout, leading to a highly textured structure. As such, successful implementation of these technologies requires a comprehensive assessment of the relationships of the key process variables, geometries, resultant microstructures and mechanical properties. The nature of this process suggests that it is often difficult to produce representative test specimens necessary to achieve a full mechanical property characterisation. Therefore, the use of small scale test techniques may be exploited, specifically the small punch (SP) test. The SP test offers a capability for sampling miniaturised test specimens from various discrete locations in a thin-walled component, allowing a full characterisation across a complex geometry. This paper provides support in working towards development and validation strategies in order for advanced manufactured components to be safely implemented into future gas turbine applications. This has been achieved by applying the SP test to a series of Ti-6Al-4V variants that have been manufactured through a variety of processing routes including EBM and investigating the structural integrity of each material and how this controls the mechanical response.

## 1. Introduction

Additive layer manufacturing (ALM) is an emerging technology that is rapidly becoming an integral method of component manufacture. The layer-by-layer process has evolved significantly over the past couple of decades and is capable of fabricating near fully dense components manufactured directly from electronic data such as computer-aided design (CAD) software. The process was initially used for rapid prototyping [1] and is now finding application towards the manufacture of near net shape structural parts [2], with significant potential cost benefit, short lead times and improved buy-to-fly-ratios [3]. Furthermore, ALM offers the opportunity to manufacture complex component designs which would not be possible with more conventional manufacturing techniques such as casting or forging. 

**Figure 1:**
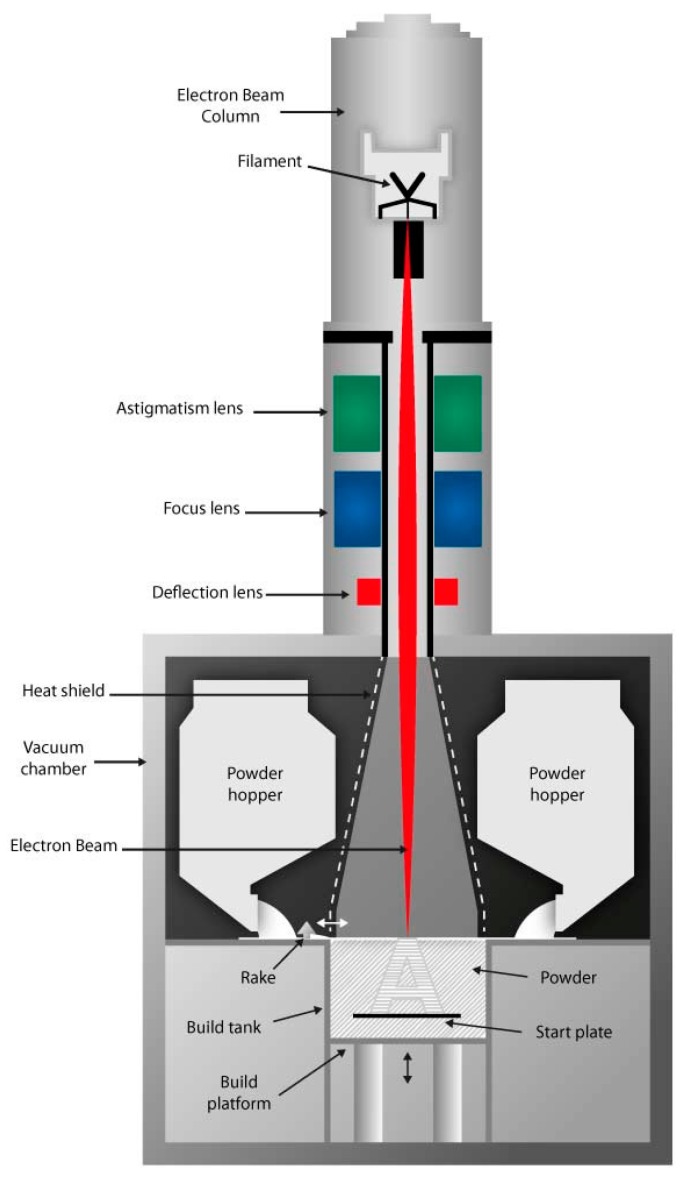
Schematic of the electron beam melting (EBM) process [4].

One method of ALM is electron beam melting (EBM), a technique in which the power from a high intensity tungsten filament sourced electron beam melts a pre alloyed metal powder upon a powder bed to a pre-programmed design. Components are built up layer by layer within a near-vacuum environment, from a powder feedstock supplied from hoppers adjacent to the build chamber as given in Figure 1 [4]. The EBM process consists of the following four stage procedure:

1. Powder raking
2. Preheating/sintering
3. Contour
4. Hatch profiling

EBM machines were first developed nearly two decades ago by Arcam AB, who remain the only commercial supplier and the systems operate under a high vacuum in order to increase the quality of the electron beam. The combined use of an electron beam within a vacuum offers an extensive range of advantages including; higher build rates due to increased penetration depths, rapid scan speeds, reduced levels of contamination and the extraction of unwanted gas from the molten material which can typically lead to residual porosity within the structure, allowing densities of greater than 99.8% to be achieved [5]. As such, materials that have a high affinity towards oxygen, such as titanium alloys, can be readily processed by EBM. Unlike many other ALM fabricating systems, the EBM process tends to occur at an elevated temperature, aided by means of a pre-heated build plate, offering the additional benefit of reducing the levels of residual stress in the material [6]. 

However despite the many benefits of fabricating components with EBM, a fundamental understanding of how the microstructure and mechanical properties evolve through the build material is of high importance. Previous literature has documented that Ti-6Al-4V, a well renowned fan blade material, built via the Arcam EBM process offers comparable tensile properties to wrought material [7,8,9,10]. Nonetheless, the results are heavily influenced by the minimal presence of microstructural inconsistencies and defects such as pores and unmelted powder particles, particularly in near surface locations. Furthermore, considering the multiple interactions of intra and inter build process variables [11] on the integrity and consistency of the final structure, traditional mechanical test approaches are deemed unsuitable for mechanical characterisation as it is difficult to produce representative test specimens for conventional mechanical test approaches which accurately replicate the microstructure, and in turn the mechanical properties of additive components. As such, miniaturised test methods provide an alternative option, whereby small disc specimens can be fabricated to characterise the properties of components with thin-wall sections and limited dimensions. One such miniaturised test approach is the small punch (SP) test.

**Figure 2:**
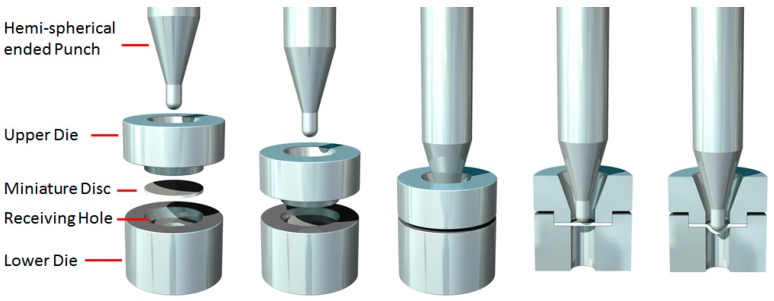
Schematic representation of the small punch (SP) test method.

The SP test initially received considerable attention in the 1980s for evaluating ductility loss in neutron irradiated materials in nuclear reactors [12,13] and for remnant life assessment of steels in the power generation industry, by the use of a scoop sample [14,15,16]. The technique has now been further developed across worldwide research laboratories and institutions to obtain creep deformation and rupture times as well as tensile fracture data across a range of material systems including steels, titanium and aluminium alloys [17,18,19,20,21]. Industry is now recognising the unique potential of the SP test for characterising the properties of small quantities of novel materials which may be seen as candidates for future aero engine applications, as larger quantities cannot be readily manufactured due to cost limitations [22]. Recent research has focused on applying the technique to more commonly recognised aerospace alloys, such as single crystal materials [23] and inherently brittle intermetallic compounds [24]. Furthermore, the technique has also been utilised in evaluating the properties of ALM materials and revealing the anisotropic nature of materials fabricated through such methods, and comparing the results to more traditional wrought equivalents [25]. Finally, the SP test also provides the capability to correlate test data generated to those produced from more conventional approaches [23] making SP testing a practical and attractive solution for many geometry related issues where material characterisation is required. The typical set up of an SP test is illustrated in Figure 2.

This research will investigate the microstructure and mechanical property relationships of EBM Ti-6Al-4V utilising the SP test method and assesses how these properties compare to those produced through more conventional manufacturing processes. The structural integrity of the different variants shall also be evaluated using advanced X-ray computed tomography, notably to determine the presence and population of residual gas porosity nearing the surface and in the internal structures. Finally, conclusions shall be drawn to address the influence of microstructure and structural integrity on the mechanical response of the Ti-6Al-4V variants.

## 2. Experimental Procedures

### 2.1. Materials

The study was carried out on three Ti-6Al-4V variants, each of which were manufactured via a different processing route, specifically: (i) cast and hot isostatically pressed (HIP); (ii) forged disk; and (iii) electron beam melted (EBM). The EBM material was built in a vertical orientation at the Manufacturing Technology Centre (MTC) in Coventry, UK on an Arcam System configured with EBM Control 3.2 Service Pack 2 software (Stockholm, Sweden). The pre-alloyed plasma atomised Ti-6Al-4V powder feedstock contained particles ranging from 45 to 100 μm in diameter. The first layer of powder was deposited onto a 220 × 300 × 10 mm^3^ thick stainless steel base plate that was pre-heated with the electron beam to 730 °C. Prior to melting each layer, the new layer of powder was preheated and sintered by scanning the beam over the cross-sectional geometry in an attempt to maintain a consistent build temperature of 730 °C throughout and reduce the effects of charging of the powder during melting. Further detail on the EBM process has previously been published in the literature [26].

#### 2.1.1. Microstructure

**Figure 3:**
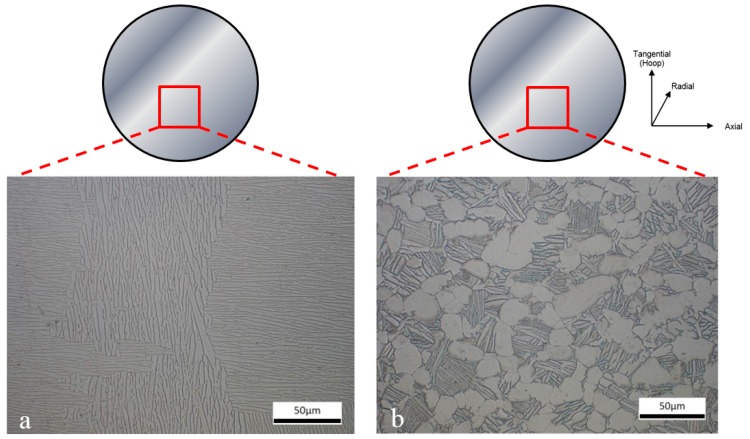
Microstructure of (**a**) Cast and HIP Ti-6Al-4V and (**b**) Forged Ti-6Al-4V small punch disc specimens prior to testing, illustrating the top surface of the SP specimen where the punch makes initial contact.

**Figure 4:**
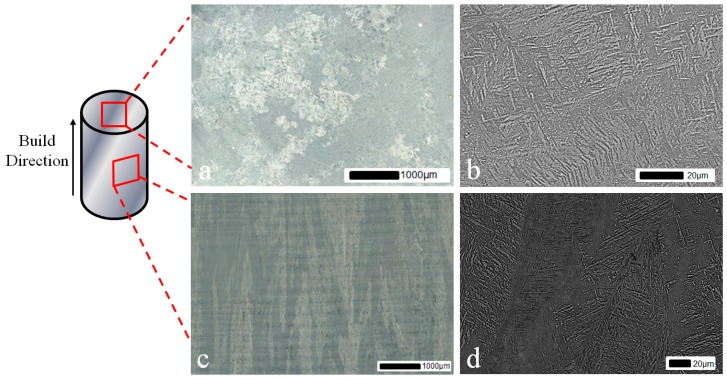
Microstructure of EBM Ti-6Al-4V (**a**) perpendicular to the build direction at low magnification; (**b**) perpendicular to the build direction at high magnification; (**c**) parallel to the build direction at low magnification and (**d**) parallel to the build direction at high magnification. Load is applied through the face perpendicular to the build direction.

Prior to testing, a microstructural evaluation of a SP miniature disc sample (9.5-mm diameter, 500-μm thickness) from each of the different Ti-6Al-4V variants was undertaken. Figure 3 illustrates the microstructure on the top surface of the SP specimens, *i.e.*, where the punch initially contacts the specimen, for the cast and HIP and forged materials. The EBM material is shown separately in Figure 4 looking at both the face parallel to the build direction and the face perpendicular to the build direction. Analysis of the microstructural features was performed using the mean linear intercept method in ImageJ, a technique used to quantify grain size by drawing a set of lines on the micrograph and counting the number of times a grain boundary intersect occurs. This was completed on both faces for the EBM material due to its highly textured microstructure. The cast and hot isostatically pressed (HIP) (Figure 3a) material has a Widmanstätten type grain morphology that consists of α and β phases with a colony size of parallel-oriented α-phase lamellar of approximately 200 μm on average and a lath width of approximately 2.44 μm. In the Widmanstätten microstructure, the α phase is formed along the prior β grain boundaries. The forged variant (Figure 3b) has a relatively equiaxed bimodal structure, with an α volume fraction of approximately 60% where the material has been extracted from the radial direction, thus analysing the tangential-axial surface. The EBM material when looking into the build direction, *i.e.*, the SP specimen surface, (Figure 4a,b) displays an ordered lamellar structure consisting of an extremely fine acicular grain morphology. This is as expected due to the thermal characteristics of the EBM process which consists of a small melt pool and rapid cooling. This has been well documented in the literature [7] and is characterised by a columnar prior-β grain structure with epitaxial β <100> crystal growth parallel to the build direction (Figure 4c,d) due to primary thermal gradients that exist in the build direction (parallel and opposite to the principal direction of heat flow on cooling). Here, the elongated grain widths were measured perpendicular to the length of the grain. Antonysamy *et al*. [27] studied the prior β-grain texture of EBM components and found that the columnar structure exhibited strong texture of <001> β normal to the deposited powder layers. It is thought that this favoured orientation could be attributed to the elongated shape of the moving melt pool and the raster pattern typically used in the EBM process. Additional microstructural measurements characterising the typical grain sizes of the different Ti-6Al-4V variants are given in Table 1, showing both the larger prior-β grain dimensions and the secondary or transformed alpha lath width internal to the prior-β grains. These are measured perpendicular to the textured grain’s largest axis. Measurements are taken as an average from over 600 calculations from multiple locations within the sample to provide a representative average.

#### 2.1.2. X-ray Computed Tomography

Following the manufacture of the SP test specimens a study was undertaken using X-ray computed tomography (CT) in order to investigate the structural integrity of the various Ti-6Al-4V materials. Previous research has found CT to be a highly effective method of determining content, size, frequency, morphology and distribution of porosity in three dimensions within metallic components [26]. CT scans were performed on each of the SP discs using a Nikon XT H 225 CT system which consists of a Nikon open type X-ray tube (Tring, UK) and a Paxscan 2520V X-ray amorphous-Si flat panel detector (Palo Alto, CA, USA). The three samples were scanned as a collective batch to ensure similitude and scan quality. Three-dimensional reconstruction of the specimens from CT data was created using two-dimensional greyscale X-ray projections, gathered while the specimen was rotated around 360 degrees. The 3D volume indicates variations in material attenuation or density which can be used to isolate features of interest. One such feature is porosity, which is typically detrimental to the mechanical properties of a given material. Scan parameters for the samples taken both before and after testing are given in Table 2. Reconstruction was achieved using Nikon CT-Pro v3.1.3 software (Tring, UK) producing a resulting voxel size of 6.7 µm for scans of the samples prior and post fracture.

**Figure 5:**
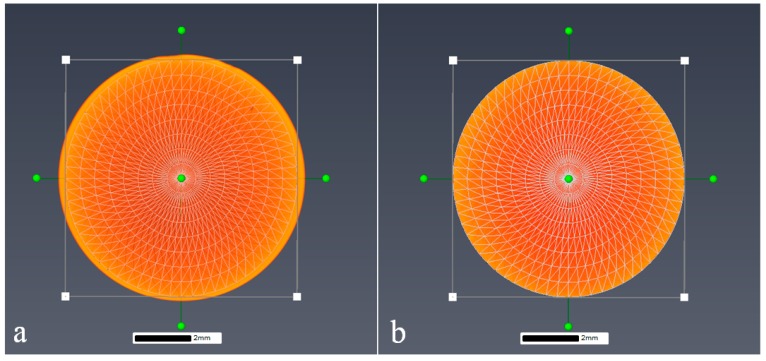
Image showing a volume render of the small punch specimen both (**a**) before and (**b**) after trimming in Avizo 9.0.

**Figure 6:**
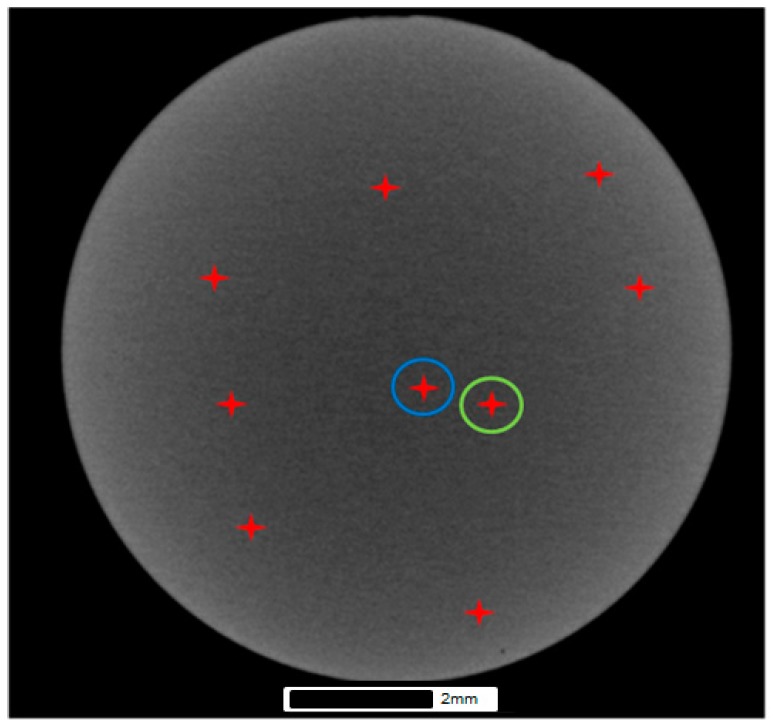
Porosity network in the EBM SP specimen using Avizo 9.0.

Feature characterisation was conducted using micro-CT with the combined use of Avizo 9.0 3D analysis software (Hillsboro, OR, USA) and VG Studio MAX 2.1 (Heidelberg, Germany). The post scan analysis was undertaken to characterise the porosity and revealed the volume fraction, orientation, size, aspect ratio and spatial distribution of the porosity where present in the structure. The porosity assessment was conducted by trimming the reconstructed scan volume to isolate the small punch volume from the surrounding outside region followed by selecting a region of interest from within the sample. The purpose of trimming the volume was to reduce the noise found at the interface between air and the sample volume due to beam interactions caused by surface imperfections at the sample surface (Figure 5). Although this is believed to remove the ability to determine and include the surface and surface breaching porosity, it serves to improve the porosity assessment of the bulk of the sample as a whole. Therefore, VG studio has been used to characterise pore size and morphology including identifying surface breaching pores whilst Avizo Fire 9.0 has been used to determine the overall characteristics of the internal pore network. This is illustrated in Figure 6, which highlights the most prominent porosity features found within the EBM disc specimen, both surface and subsurface. From this figure, a total of 9 of the most prominent pores have been highlighted, from which two (circled blue and green) fall within the cross section of the specimen where the 2.5 mm diameter punch indentation will occur and are thus expected to be located close to or within the SP fracture region. As a result, they are further assessed in the final section of the paper.

**Figure 7:**
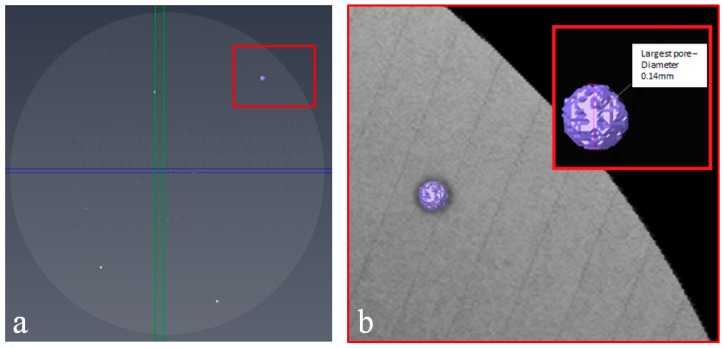
Images of (**a**) 3D volume render and (**b**) the largest porosity feature in the EBM disc using VG studio.

Figure 7a illustrates the 3D volume render of the EBM disc after greyscale thresholding within VG studio and indicates the size of porosity through the structure. Figure 7b indicates the largest pore in the specimen, which measures approximately 0.14 mm in diameter from within the bulk of the material. Due to its size it is believed that this feature is a byproduct of the manufacturing process rather than being the result of residual gas pores originating from the manufacture of the powder as this size falls considerably outside the powder size distribution (45–100 µm). The overall porosity data is summarised in Table 3, which contains the porosity calculations for each of the three Ti-6Al-4V variants.

As Table 3 shows, in the EBM disc, a number of internal inconsistencies were found from the reconstructed data which can be attributed to porosity. In order to develop a fair interpretation of the material a certain number of such features must be discounted. As the size of any feature nears closer to the voxel size, the certainty of its size, morphology or even existence as a feature rather than being attributed to noise becomes smaller. Beam hardening effects may be reduced using a physical pre-filter, but it has been found that the SP disc geometry naturally yields a number of smaller features around the single voxel size. Although such artifacts may represent porosity, the risk of noise artifacts being attributed as porosity has been reduced by discounting features lower than a certain threshold. Based on previous research, a minimum feature size has been considered to be a minimum of 2 × 2 × 2 (8 voxels) [26], assuming features of porosity from the perspective of a cube. As such, the EBM material density was calculated to be 99.97% using an 8 voxel cut-off threshold.

In comparison, both the cast and HIP and forged variants were found to have no internal inconsistencies at this length scale and no observed porosity content, hence making them essentially fully dense. This calculation, however, is limited by the voxel size of approximately 7 µm, so the accuracy and reliability of measured features beyond this limit is reduced, but the CT process can be used for detecting and measuring larger features and shows potential for accurately highlighting location, spatial distribution and morphology which may contribute towards anomalous material responses especially from smaller sample geometries.

### 2.2. Small Punch Testing

The SP tensile test was used in this research to assess the tensile properties of the different Ti-6Al-4V processing variants. SP miniature disc specimens of EBM and forged Ti-6Al-4V were extracted from the threaded ends of conventional tensile specimens which had been turned down on a CNC lathe to a diameter of 9.5 mm. Cast and HIP Ti-6Al-4V SP specimens were extracted from a larger piece by means of wire electrical discharge machining (EDM). In either case the resulting cylinders were sectioned into segments approximately 700–800 µm in thickness using a Secotom-10 cutting wheel facility (Ballerup, Denmark) prior to being ground down and polished to the required thickness of 500 µm ± 5 µm. This is achieved by using successively finer grades of silicon carbide abrasive paper, the final grade being 1200 grit, in accordance with the recommendations of the European Code of Practice (CoP) for Small Punch testing [16]. Disc thickness is measured at 5 locations around the periphery and the center of the specimen to ensure uniformity of thickness, again conforming to the CoP [16].

**Figure 8:**
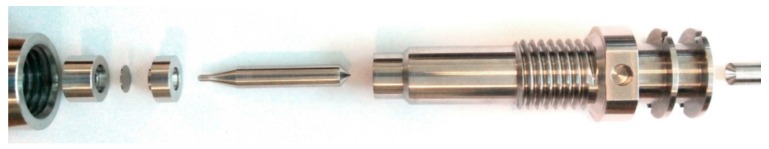
Small Punch test configuration.

To perform the experiments, a bespoke high temperature SP test jig (Figure 8) has been designed for location within the load train of a universal, servo-actuated 100 kN electric screw test frame in order to test miniature disc specimens at elevated temperatures under relatively high loads. This experimental facility has the potential to perform either small punch creep (constant load) or small punch tensile (constant displacement) tests on various material systems across a range of temperature regimes. In either test configuration, the punch head displacement is measured by two linear variable displacement transducers (LVDTs) situated in parallel via a dual arm extensometer cage attached to the jig. The SP miniature disc sample is clamped within an upper and lower Nimonic-90 die to prevent the specimen being drawn into the receiving hole. The applied load is transferred to the specimen via a 2.5 mm diameter hemispherical ended Nimonic-90 punch, perpendicular to the top surface of the disc, through a receiving hole of 4 mm diameter, conforming to the recommendations of the CoP [16].

## 3. Results and Discussion

### 3.1. Small Punch Test Results

**Figure 9:**
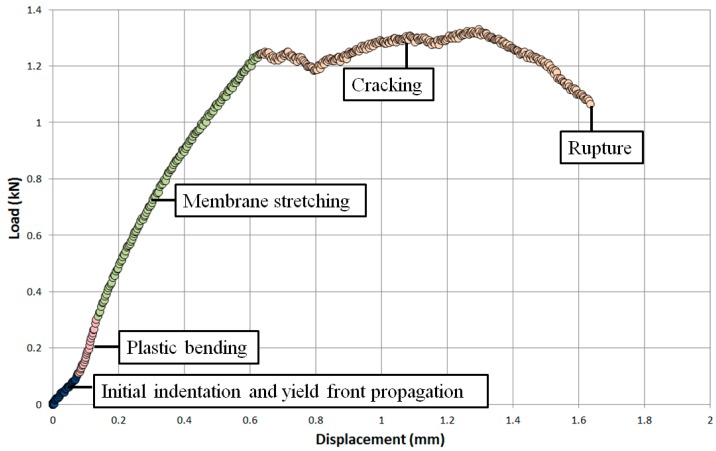
Load-displacement relationship of a SP tensile test on a cast and HIP Ti-6Al-4V specimen.

Figure 9 shows the typical small punch load-displacement curve obtained for the cast and HIP material at a temperature of 20 °C and displacement rate of 1.2 mm·min^−1^. The response for this material is consistent to the behaviour previously published in the literature [15], where the initial region of the curve demonstrates a small period of initial indentation and compression upon loading of the punch, which is quickly followed by material yield. As the miniature disc specimen plastically deforms under the applied load, the material experiences an extended phase of plastic bending and membrane stretching. The material then achieves the first load peak and from thereafter, the disc begins to gradually thin and crack, leading to the onset of rupture. For each small punch tensile test described throughout this research, the displacement at the onset of failure is defined as the punch displacement at 20% load drop after the maximum load, in accordance with the CoP [16]. Each of the stages of deformation are highlighted in Figure 9.

**Figure 10:**
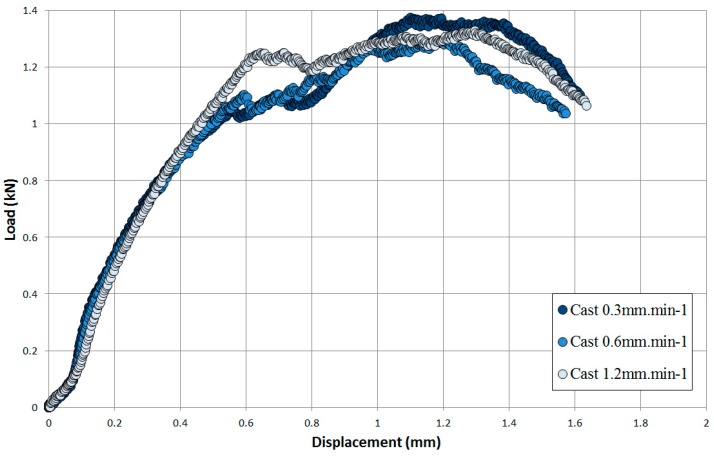
Effect of displacement rate on cast and HIP Ti-6Al-4V.

In order to determine whether the rate of displacement had an influence on the deformation behaviour of Ti-6Al-4V, a series of SP tensile tests were performed on the cast and HIP material under a variety of displacement rates. The CoP [16] recommends a displacement rate between 0.2 mm·min^−1^ and 2 mm·min^−1^ to ensure a level of consistency for data generation. As such, the displacement rates of 0.3 mm·min^−1^, 0.6 mm·min^−1^ and 1.2 mm·min^−1^ were employed for this research. Figure 10 illustrates that the rate of displacement appears to have minimal effect in the early stages of deformation. For each rate, the periods of initial indentation, yield front propagation and membrane stretching are highly comparable. However, as each test approaches the first load peak, a clear discrepancy in response develops as the test performed at the fastest rate (1.2 mm·min^−1^) achieves the highest load, thus giving the stiffest response, followed by the 0.6 mm·min^−1^ and 0.3 mm·min^−1^ tests respectively. These results are in agreement with the trends observed in more conventional mechanical test approaches where a series of uniaxial tensile tests on Ti-6Al-4V performed under different strain rates found that the yield stress and ultimate tensile strength (UTS) of the material increase with a faster strain rate [26]. After this load peak, the final stages of deformation are less uniform as each of the discs start to crack in a more uncontrolled nature until final rupture occurs.

**Figure 11:**
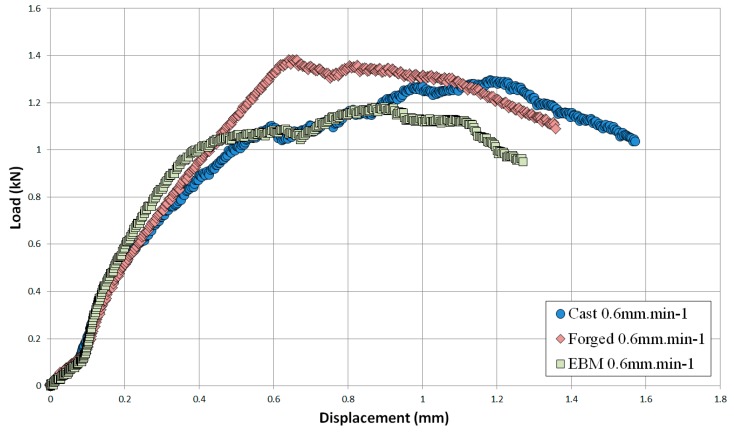
SP tensile response of EBM, cast and HIP and forged Ti-6Al-4V variants at a displacement rate of 0.6 mm·min^−1^.

SP tensile tests were then performed on each of the Ti-6Al-4V variants to directly compare their respective tensile responses. A consistent displacement rate of 0.6 mm·min^−1^ and test temperature of 20 °C was employed for each experiment and the load-displacement results are presented in Figure 11. The figure shows that the cast and HIP and EBM discs have a similar response during the initial loading stage to the point where membrane stretching begins. The forged material however appears to accumulate a higher proportion of load over this period indicating that this variant exhibits a stiffer response. This could be attributed to the reduced grain size of the forged material when compared to the other variants (as previously stated in Table 1) and this follows the expected trend as proposed by Hall-Petch. As the first load peak is then approached, the forged material achieves the highest load (1.4 kN), whereas the cast and HIP and EBM specimens have a similar response, reaching an initial peak load of around 1.1 kN after 0.55 mm of displacement. From this stage, each of the material variants start to undergo cracking and it can be seen that the ultimate peak load of the EBM material does not appear to significantly increase from the initial peak. This is consistent with the behaviour of the forged material which is unable to reach a maximum load in excess of the first, whereas the cast and HIP variant achieves an ultimate load peak which is considerably higher than the first, occurring over a relatively larger level of displacement. In each case, the increased ductility in these two materials is observed in comparison to the EBM counterpart. In the EBM material, it is shown that once cracking commences, at a lower first peak than the forged material, the debit in strength in the material is too great and unrecoverable for the disc to manage a second load peak in excess of the first, unlike the cast and HIP material, and thus fails under a lower level of overall displacement along with a lower ultimate load.

### 3.2. Small Punch Fractography

**Figure 12:**
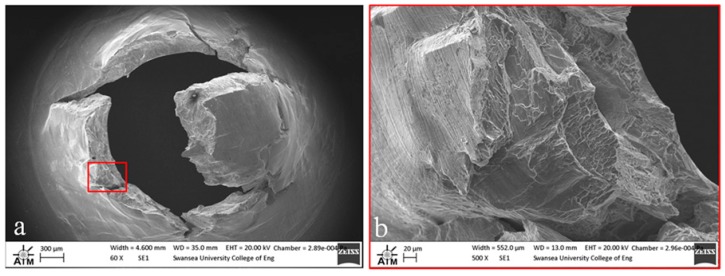
SEM images of fractured SP disc of the cast and HIP Ti-6Al-4V material showing (**a**) overall fracture surface and (**b**) typical rupture behaviour.

**Figure 13:**
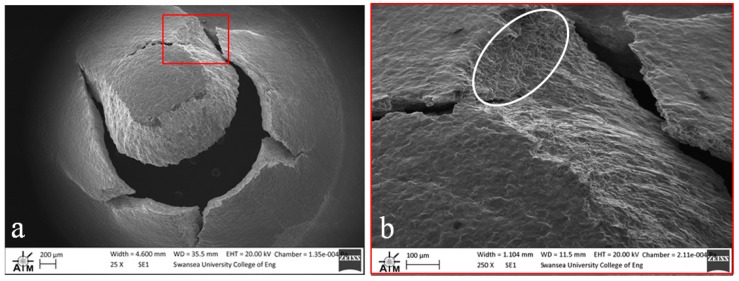
SEM images of fractured SP disc of the forged Ti-6Al-4V material showing (**a**) overall fracture surface and (**b**) typical rupture behaviour.

**Figure 14:**
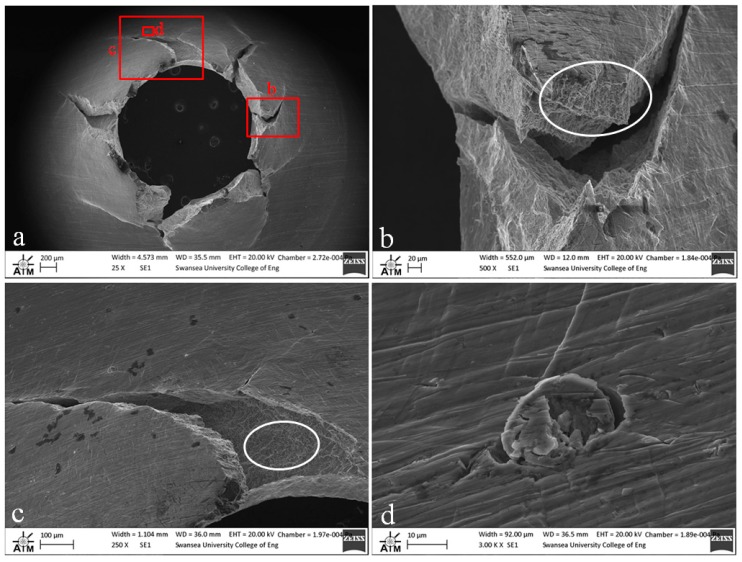
SEM images of fractured SP disc of the EBM Ti-6Al-4V material showing (**a**) overall fracture surface; (**b**) typical rupture behaviour; (**c**) separation of melt layers and (**d**) processing anomaly.

Fractography of the tested small punch disc specimens was undertaken on a Zeiss EVO LS25 Scanning Electron Microscope (SEM). From the images given in Figure 12, Figure 13 and Figure 14, it is evident that the mode of deformation changes across the various materials. While each of the miniature disc samples exhibit significant plastic deformation, as illustrated by the dome type appearances in the aerial views, the extent of ductility and radial ‘star’ type cracking differs. Figure 12 presents the fracture behaviour of the cast and HIP disc, and shows that the material appears to elongate to a greater extent than the forged and EBM variants, and also exhibits a lesser number of dominant cracks, thus illustrating the most ductile response. This is in accordance with uniaxial tensile results previously documented [28] on the same material, whereby a ductile transcrystalline dimple type fracture is typically observed. Similarly, the forged variant has a relatively ductile response (Figure 13a) with the fracture surface dominated by elongated intergranular dimples (as highlighted by the white circle in Figure 13b) that occur along the continuous α layers at β grain boundaries. This again complements the findings by Lutjering [29] who found that the mechanical properties of conventionally processed alloys are generally controlled by the α colony size, whether in terms of lath width or grain size. This trend reflects the SP test results in which the cast and HIP and forged materials exhibit more evidence of ductile deformation, whereas such features are less prominent in the EBM variant.

In contrast, the fracture appearance of the EBM disc is more consistent with the behaviour that would be expected for a more brittle type material. This is illustrated in Figure 14a, where the number of dominant radial cracks has increased and the specimen appears to have failed in a more transgranular type fashion as highlighted by the relative smoothness of the fracture surface as the crack path travels through a number of grains (Figure 14b). The fracture surface is not entirely devoid of ductile features however, as highlighted by the white circles in Figure 14b,c. The images show a quasi-cleavage type fracture with some presence of ductile dimple tearing and a distinct number of fine and deeper dimples can be seen on the surface. This behaviour is similar to uniaxial results presented in the literature on EBM material [30] where deformation typically exhibits a mixed mode of ductile and brittle fracture. Other contributing factors to such behaviour are given in Figure 14c,d, which appears to indicate the separation along a plane that is parallel to the layers (Figure 14c) and what is likely to be a partially sintered powder particle (Figure 14d), two features that could have an influence on the performance and integrity of an additive material, depending on the application.

**Figure 15:**
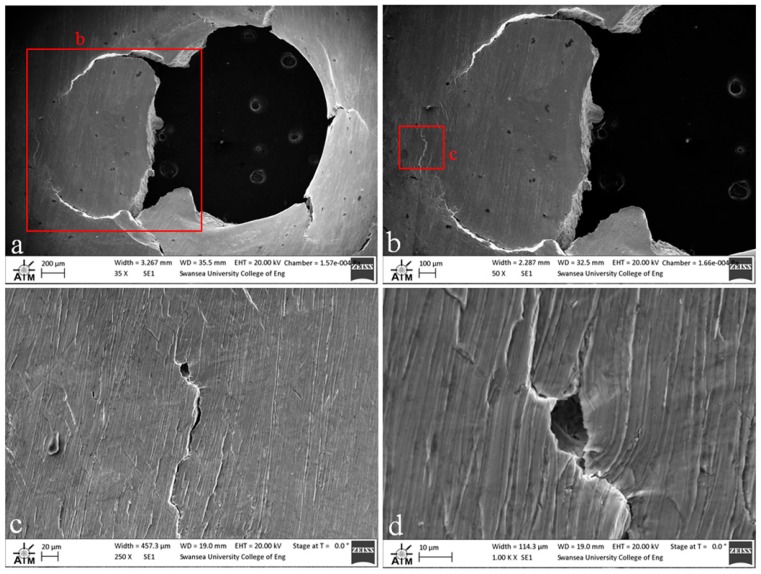
SEM images of the reverse side of the fractured EBM SP disc (**a**) overall fracture surface; (**b**) indication of the dominant crack path (**c**,**d**) typical surface breaking porosity.

These findings were further evidenced when analysis was extended to the reverse side of the EBM disc. Upon inspection (Figure 15), a series of prominent porosity type features were evident on the surface and a radial crack path could be identified where it appears to interact with localised porosity (Figure 15b–d). As previously mentioned, the main source of gas porosity is generally believed to be a result of the manufacturing process. This, in combination with gas that may be found within hollow powder particles, can contribute to gas pockets that are unable to escape during the build of successive layers, altogether leading to a reduction in the tensile performance of the material and thus complementing the results found in this research.

Subsequently, to finalise the holistic understanding of each of the fracture mechanisms taking place, each of the tested SP specimens where mounted, polished and analysed under an SEM in order to reveal the microstructure of the followed crack paths in each of the Ti-6Al-4V variants.

**Figure 16:**
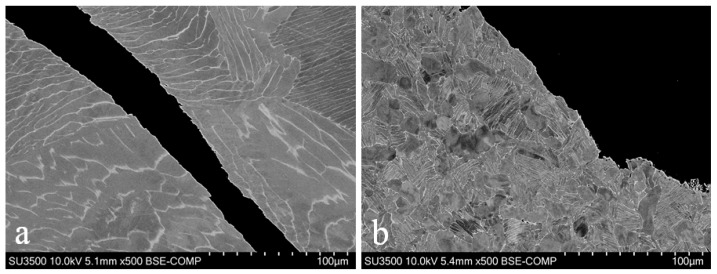

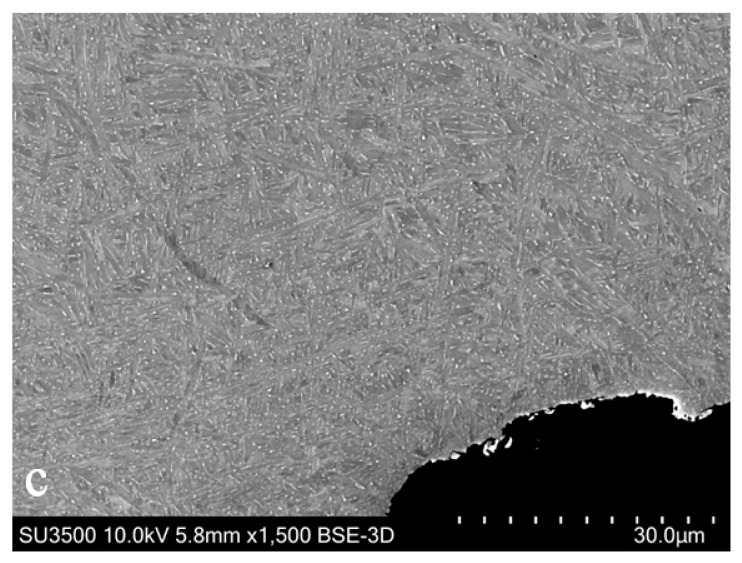
SEM images of the microstructural crack path through (**a**) cast and HIP (**b**) forged and (**c**) EBM disc specimens.

In the cast and HIP material, the radial dominant cracks emanating from the center of the disc can be seen to be very smooth in nature thus highlighting the transgranular nature of the fracture mechanism with the crack propagating across the alpha laths relatively unhindered (Figure 16a). On the other hand, the forged material clearly demonstrates the crack path following an intergranular fracture mechanism through the bi-modal microstructure, propagating along grain boundaries (Figure 16b). The microstructure crack paths corroborate the interpretations of the SP fracture surfaces for the cast and HIP and forged Ti-6Al-4V variants. The crack behaviour observed in the EBM material appears to be slightly more complex, where the fracture may be deemed as mixed mode, the crack can be seen to occur preferentially along the needle like α lamellae in a brittle transgranular type manner, as well as evidence of the crack following the prior β grain boundaries. Overall, the EBM crack growth behaviour could be considered to be relatively instantaneous in that numerous micro cracks can be seen to be protruding into the material from the primary crack path.

### 3.3. Post Test X-ray CT

**Figure 17:**
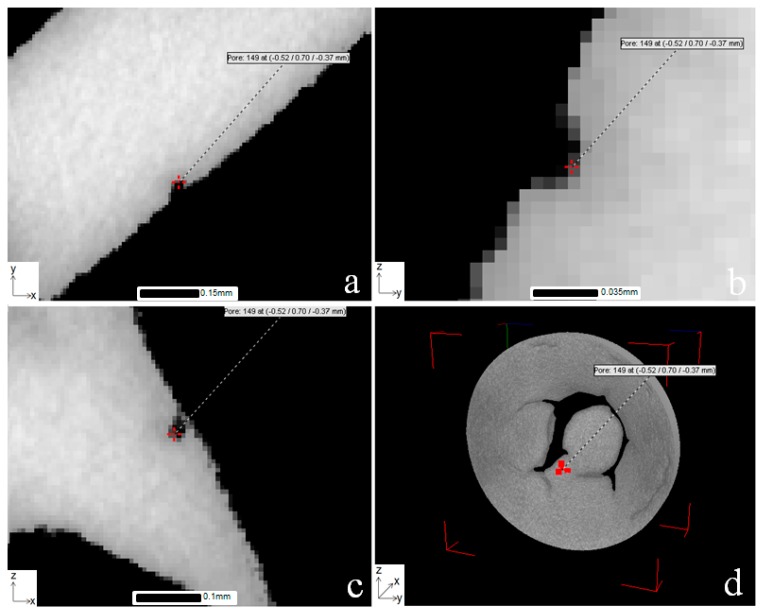
VG Studio representation of a surface breaching pore within the bulk material of the fractured EBM sample in (**a**) *z* direction; (**b**) *x* direction; (**c**) *y* direction and (**d**) 3D volume render of the isolated deformation region.

Post test CT analysis has been conducted to ascertain whether the prominent porosity feature found within the EBM fracture region (highlighted within the blue circle in Figure 6) had coincided with the crack path. Pre-test and post-test CT scans have been compared and SEM images have been used to illustrate the findings. By aligning the post test scan volume sample to the same orientation as the pre-test orientation, it was found that three pores of interest were close to the fracture surface. After an initial assessment, the suspected surface breaching pore shown in Figure 15 was found as a feature within the post-test scan. However, the pore was too close to the limitations of the voxel size to confirm its presence. The second, more prominent surface breaching pore, measuring approximately 40 µm (Figure 17) was traced back to the fracture region. Figure 17 highlights the location of the pore on the three orthogonal planes as well as the location in the volume render.

**Figure 18:**
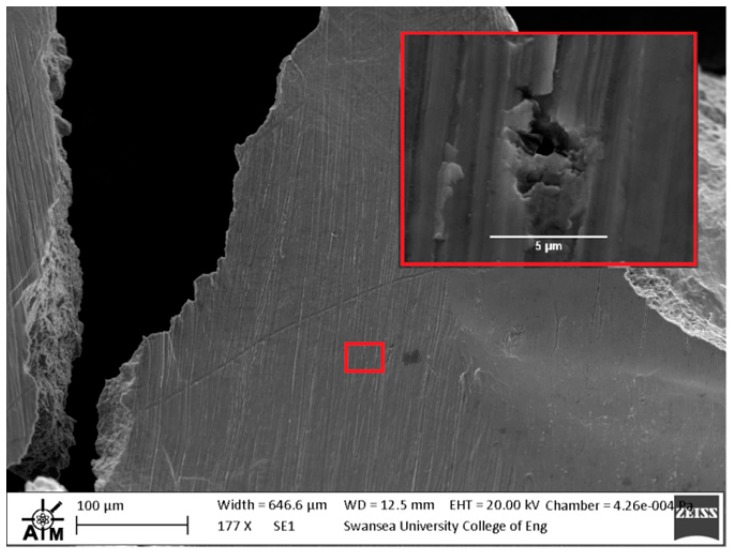
SEM image of the surface breaking pore highlighted in Figure 17 by XCT.

**Figure 19:**
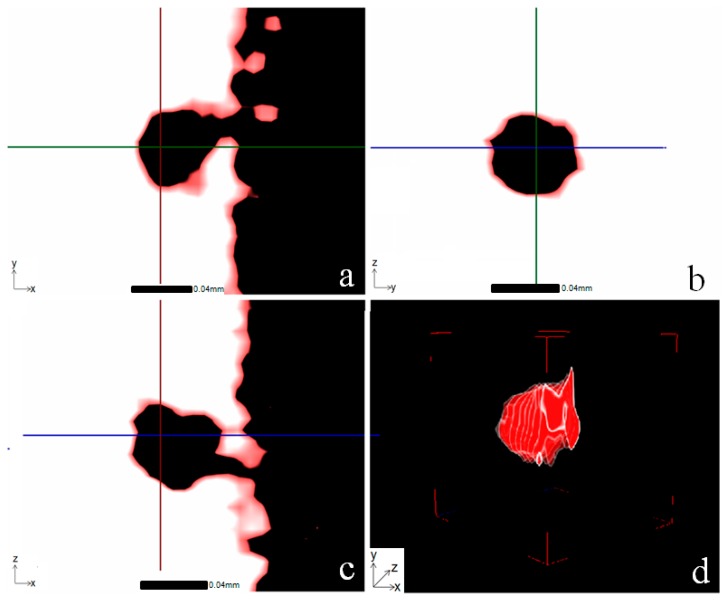
Images of the most prominent surface breaching pore in the EBM disc specimen closest to the fracture region (**a**) *z* direction; (**b**) *x* direction; (**c**) *y* direction and (**d**) full 3D representation, as given in VG studio.

The location was then correlated using SEM, as shown in Figure 18. This pore is then fully visualised in Figure 19, which depicts the same porosity feature in the *x*, *y* and *z* directions together with a full 3D representation of the feature. As shown in the figure, the pore breaches the surface and highlights the importance of adopting a 3D imaging technique to visualise such features since the size of the pore at the surface appears considerably smaller in volume under SEM analysis compared to the more accurate representation produced using CT. Comparison between pre-scan and post-scan CT data indicates that the feature has reduced slightly in size and the pore opening does not appear to contribute to any crack initiation, likely due to its location meaning it observes the initial compressive load applied in the small punch test rather than the plastic deformation and membrane stretching. Indeed the initial scan suggested that the pore was present but a proportion of it was hidden due to the grinding preparation of the disc. VG Studio software measurement of the XCT data revealed that the reduction in size was around 5 µm, but the accuracy of this measurement is perhaps limited as result of the voxel size.

The third pore of interest detected within the bulk of the material from the pre-test scan data, measured at around 35 µm within the expected deformation field (as highlighted by the green circle in Figure 6) could not be identified from the CT data. Due to the size and location it was considered to be the easiest to locate and therefore it is considered that it may have in fact been incorporated into the fracture surface. Nevertheless, no evidence of its presence could be found using CT and SEM post-test, potentially due to the depth and nature of the crack.

It was originally suspected that the pore distribution structure identified from the pre-test scan may become suggestive in predicting the final crack morphology. This would be expected provided that the porosity was significant enough to either produce weaker regions or a significant drop in material cross section to increase the localised stresses and thus induce a preferential crack path. Although small pores and features of interest such as unmelted powder particles have been noticed along the SP tensile fracture surface, there seems to be an overall inconsistency with such a trend, particularly for this sample. Where subsurface or surface breaching pores have been noted on the fracture surface, there has been no clear evidence of initiation due to such features. In some cases, as with Figure 16, the features have been noticed at the middle to the end of the crack, suggesting that the initial higher energy release during fracture is high enough to avoid coalescence of the porosity network. However, in the case of this sample and the pores identified, the primary pore network does not appear to significantly influence the crack morphology. 

Although at this stage the significance of the porosity network seems to have diminished, it is important to consider the relationship between pore and sample size. Considering the effective thin walled structure of the SP sample, a relatively large bulk or subsurface pore (Figure 7) placed under tensile loading rather than compressive loading may in fact lead to a reduction in expected mechanical properties. Thus, it has been demonstrated that CT can play a key role in the analytical procedure of SP miniature disc specimens where it offers a capability of revealing potential inconsistencies in the geometry which may then lead to anomalous data amongst a larger assessment of mechanical properties of ALM components.

## 4. Conclusions

In this study, the structural integrity of cast and HIP, forged, and electron beam melted (EBM) Ti-6Al-4V has been investigated through utilising the SP test technique in conjunction with X-ray computed tomography (CT). Results have shown that for each material, it appears that the small punch fracture behaviour is consistent with the extensive research gathered for uniaxial tensile deformation properties on the numerous processing routes of Ti-6Al-4V, where the SP tensile test can successfully characterise and rank the responses of the various materials. Likewise, this trend is seen in the post-test fractography where the cast and HIP and forged materials contained a number of features characteristic of a ductile mode of deformation, such as transcrystalline dimples and microvoid formation, whereas these features are less prominent in the very fine microstructure of the EBM variant. This relationship is further corroborated by X-ray CT analysis, which is able to identify and quantify the presence of gas porosity and other processing anomalies in the EBM material.

## Figures and Tables

**Table 1:** Microstructural measurements of Ti-6Al-4V variants.

Measurement	Cast	Forged	EBM
Mean Linear Intercept (1/µm)	0.0050	0.0778	0.0041
Prior-β grain width (µm)	201	13	246
Transformed alpha width (µm)	2.4410	1.6868	0.7867

**Table 2:** Computed tomography (CT) scan parameters of EBM small punch disc specimen before and after testing.

Sample	Accelerating Voltage (kV)	Current (μA)	Filter	Filter Thickness (mm)	Number of Projections	Frames per Projection	Exposure (ms)	Target Metal
Pre Test	90	459	Ti	2.1	3016	2	1000	W
Post Test	105	490	Ti	2.1	3016	1	1000	W

**Table 3:** Porosity measurements of Ti-6Al-4V variants (minimum voxel size of 6.7 µm).

Measurement	Cast	Forged	EBM
Maximum Pore Diameter (μm)	n/a	n/a	136.7
Minimum Pore Diameter (μm)	n/a	n/a	16.8
Average Pore Diameter (μm)	n/a	n/a	19.6
Number of features	0	0	897
